# 

**Published:** 1892-06

**Authors:** 


					CONTENTS.
Extra-Uterine Pregnancy.
By A. E.
Aust Lawrence, M.D. ...
73
Hay Fever and Hay Asthma.
By
P. Watson Williams, M.D.
84
Health Resorts in the West of
England and South Wales.
YI.?Tenby.
By J. G. Lock
97
Progress of the Medical Sciences:
Medicine.
By J. Michell Clarke,
M.B.
107
Surgery.
By W. H. Harsant ..
113
Obstetrics.
By L. M. Griffiths
117
Laryngology and Rhlnology.
By
Barclay J. Baron, M.tf.
123
Reviews of Books
127
Notes on Preparations for the Sick
142
Meetings of the Bristol Medico-
Chirurgical Society
Hi
The Library of the Bristol Medico-
Chirurgical Society   146
Presentation to Sir George
Buchanan
149
Jefferson Medical College ...
150
Scraps
ISO

				

